# Evaluating the efficacy of valproic acid in alcohol use disorder: a systematic analysis of clinical trials from ClinicalTrials.gov

**DOI:** 10.3389/fphar.2025.1503035

**Published:** 2025-04-25

**Authors:** Fahad S. Alshehri

**Affiliations:** Department of Pharmacology and Toxicology, College of Pharmacy, Umm Al-Qura University, Makkah, Saudi Arabia

**Keywords:** alcohol use disorders, valproic acid, clinical trials, systematic analysis, alcohol dependence, anticonvulsant therapy

## Abstract

**Background:**

Alcohol use disorder (AUD) represents a significant global health burden, characterized by high relapse rates and limited treatment options. Valproic acid, primarily used as an anticonvulsant and mood stabilizer, has been suggested as a potential therapeutic agent for AUD, particularly in patients with coexisting psychiatric conditions. This study systematically analyses clinical trials from ClinicalTrials.gov to evaluate the efficacy of valproic acid in treating AUD.

**Methods:**

A systematic search of ClinicalTrials.gov was conducted to identify clinical trials involving valproic acid in the management of substance use disorder (SUD). A total of 3,822 studies related to SUD were initially identified. Screening for anticonvulsant use narrowed this to 96 trials, and four completed studies specifically involving valproic acid and AUD were included in the final analysis. Key outcomes related to relapse rates, substance use reduction, mood stabilization, and withdrawal symptoms were examined.

**Results:**

The included studies focused on various conditions, including alcohol dependence, bipolar disorder with substance abuse, traumatic brain injury with alcohol use, and medication-overuse headache. Valproic acid demonstrated potential benefits in reducing alcohol consumption, stabilizing mood, and managing withdrawal symptoms in specific subpopulations. However, relapse rates remained high in some trials, indicating limited long-term efficacy. Secondary outcomes showed improvements in psychiatric symptoms, though adverse effects such as sedation and gastrointestinal disturbances were noted.

**Conclusion:**

Valproic acid shows potential as a therapeutic option for managing AUD, particularly in individuals with coexisting psychiatric conditions or complex clinical profiles. While the drug showed some efficacy in reducing substance use and stabilizing mood, the overall impact on long-term abstinence remains uncertain. Further research is needed to better define the role of valproic acid in AUD treatment and to identify patient populations that may benefit most from its use.

## Introduction

Substance use disorder (SUD) represents a critical global health challenge with important clinical, social, and economic implications (Taylor et al., 2023; Bush et al., 2016). According to the World Drug Report 2021 by UNODC, an estimated 275 million people aged 15–64, or 1 in 18 globally, used substances in 2023, with 13% experiencing SUD (WHO, 2023). SUD is frequently associated with high rates of psychiatric and somatic comorbidities, complicating treatment and reducing the quality of life for affected individuals (Gardvik et al., 2021; Schuckit, 2006; Skarstein et al., 2023). The economic burden is significant, with annual treatment costs for SUD in hospitals in the United States alone reaching US$13.2 billion (Peterson et al., 2021). Despite the significant public health impact, current treatment strategies for SUD remain limited and often ineffective, resulting in poor long-term outcomes and high relapse rates.

Pharmacological interventions are important in the management of SUD, particularly for specific substances such as alcohol and opioids (Han et al., 2021; Ray et al., 2020; Bell and Strang, 2020). For example, medications like naltrexone, acamprosate, and methadone have been widely utilized to manage alcohol and opioid use disorders (Bell and Strang, 2020; Edinoff et al., 2021; Kirchoff et al., 2021; Maisel et al., 2013; Soyka, 2015). However, the efficacy of these treatments differs among individuals, and relapse rates are still high. Therefore, recent studies have shown that medications such as valproic acid could have potential for the treatment of AUDs (Romão et al., 2022; Ahmed et al., 2019; Singh et al., 2021). Valproic acid, a mood stabilizer commonly used in the treatment of epilepsy and bipolar disorder, exerts its effects through multiple mechanisms, including the modulation of gamma-aminobutyric acid (GABA) levels, inhibition of voltage-gated sodium channels, and suppression of histone deacetylases (HDACs) (Rahman et al., 2020; Tomson et al., 2016).

Emerging evidence suggests that valproic acid may offer therapeutic potential for certain subpopulations of individuals with alcohol use disorder (AUD), particularly those with AUD and comorbid psychiatric conditions (Salloum et al., 2005; Weiss et al., 2023; Carli et al., 2023). Clinical trials investigating the efficacy of valproic acid as a treatment for AUD have produced mixed but promising results, highlighting the need for further exploration. For instance, studies have demonstrated that valproic acid may reduce alcohol consumption and improve mood stability in patients with AUD and co-occurring mood disorders, suggesting a dual benefit in this challenging population (Litten et al., 2016). Moreover, valproic acid’s potential to modulate the neural circuits involved in reward processing and impulse control positions it as a candidate for addressing some of the core neurobiological dysfunctions observed in AUDs in animals (Al Ameri et al., 2014; Bass et al., 2020).

This review systematically evaluates the efficacy of valproic acid as a treatment option for AUD, particularly in populations with coexisting psychiatric conditions. Given the high relapse rates and limited effectiveness of existing treatments for AUDs, this analysis aimed to assess the clinical potential of valproic acid by reviewing completed trials from ClinicalTrials.gov. Thus, this review helps to understand valproic acid’s role in managing AUDs and identifies patient subgroups that could benefit most from its therapeutic effects.

## Methods

A systematic analysis was conducted to identify clinical trials evaluating the use of valproic acid in SUD. The primary data source was ClinicalTrials.gov, a comprehensive registry of clinical studies. The search strategy was designed to capture all relevant trials involving anticonvulsants in the context of SUD.

### Study identification and screening

Records Identified: A total of 3,822 studies were initially identified from ClinicalTrials.gov by searching for trials related to SUD on September 2024. Screening for Anticonvulsant Use: From the identified records, 96 studies involved the use of anticonvulsant medications in the treatment of SUD. Selection of Valproic Acid Trials: Further screening identified four completed clinical trials that specifically investigated the use of valproic acid as an intervention for AUD. These trials met the inclusion criteria of focusing on completed studies with reported outcomes.

### Inclusion and exclusion criteria

The inclusion criteria were: (1) studies investigating the use of valproic acid or its derivatives as a primary intervention, (2) trials targeting populations with a diagnosis of SUD or related conditions such as alcohol dependence, bipolar disorder with substance abuse, or withdrawal syndromes, and (3) trials that were completed and had reported primary and/or secondary outcomes. Studies were excluded if they were ongoing, withdrawn, unknown, or did not specifically assess valproic acid as part of the intervention.

### Outcome measures and data extraction

Primary outcomes included efficacy measures such as relapse rates, changes in substance use patterns, and improvements in mood or psychiatric symptoms. Secondary outcomes focused on withdrawal symptoms, mood stabilization, and any reported adverse effects associated with valproic acid treatment. Data were extracted from the identified trials, including information on study titles, statuses, conditions treated, specific interventions, and reported outcomes.

## Results

**FIGURE 1:**
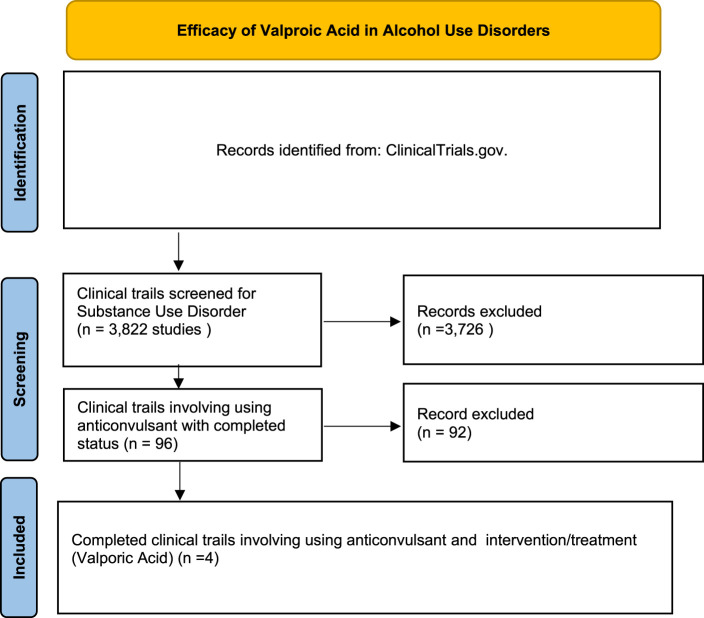
PRISMA flow diagram of study selection process.

A systematic search of ClinicalTrials.gov showed 3,822 clinical trials related to SUD. Of these, 96 studies involved the use of anticonvulsant medications. Further screening identified four completed trials specifically investigating valproic acid as an intervention for AUDs. The included studies varied in populations, interventions, and primary outcomes, focusing on conditions such as alcohol dependence, bipolar disorder with substance abuse, traumatic brain injury (TBI) with alcohol use, and medication-overuse headache. The study selection process is detailed in the PRISMA flow diagram (Figure 1), and the characteristics of each study are summarized in Table 1.

**TABLE 1:** Summarizing the key characteristics of the clinical trials, including study titles, status, conditions, interventions, primary outcomes, and completion dates.

Study title	Status	Conditions	Interventions	Primary outcomes	Location	Completion date	ClinicalTrials.gov ID
Divalproex ER vs Risperidone for Bipolar Disorder With Comorbid Substance Use Disorder	Completed	Substance Abuse|Bipolar Depression	DRUG: divalproex sodium ER|DRUG: risperidone	The primary objective is to evaluate the safety and efficacy of divalproex extended release (ER) compared to risperidone in the treatment of bipolar disorder with comorbid substance use disorder	United States	2007–02	NCT00203528
Treatment Strategy for Alcohol Use Disorders in Veterans With TBI	Completed	Alcohol Dependence	DRUG: Valproate|DRUG: Naltrexone	Time to Relapse to Heavy Drinking as Defined by Having 5 or More Drinks in a Sitting for Men and Will be Assessed Using the Time Line Follow Back for Recent Drinking Method. A Structured Questionnaire Will Review Alcohol Consumed on the Previous Week., 24 weeks	United States	2015–06	NCT01342549
Valproate for Mood Swings and Alcohol Use Following Head Injury	Completed	Traumatic Brain Injury (TBI)|Alcoholism	DRUG: divalproex sodium|DRUG: Placebo	Severity of Affective Lability Based on Shortened Agitated Behavior Scale, Severity of affective lability was measured using a shortened version of the Agitated Behavior Scale (ABS). The ABS is used to assess the nature and extent of present agitation. Eight items from the 14-item scale were used, which measured the presence and severity of various affective lability symptoms including: short attention span, impulsivity, uncooperative behavior, violent tendencies, restlessness, rapid or excessive talking, sudden changes in mood, and easily initiated or excessive crying and/or laughter. Each of the eight items was scored using a 1–4 Likert scale, where 1 stands for absence of symptom and 4 stands for presence to an extreme degree. The minimum possible score for this measure was 8, and the maximum possible score was 32. Due to the nature of the measure, a lower score indicated less severe affective lability, while conversely higher scores indicated more severe affective lability. The mean of scores for weeks 2 through 8 for each group were reported., Weeks 2 through 8	United States	2016–06	NCT01760785
Medication-overuse Headache (MOH): Withdrawal or Use of Preventative Medications Directly?	Completed	Headache	DRUG: Betablockers or other preventive drugs based on primary headache type	Headache Days, Change in Headache days per month, 5 months	Norway	2007–12	NCT00159588

The primary outcomes assessed across the trials included relapse rates, reductions in substance use, and improvements in mood or psychiatric symptoms. One trial compared divalproex extended release (ER) with risperidone in patients with bipolar disorder and substance abuse, reporting reductions in mood instability and substance use. Another study examined valproate in veterans with alcohol dependence and comorbid psychiatric conditions, which reported a decrease in episodes of heavy drinking and improvements in mood stability. A separate trial investigated valproate use in individuals with TBI and alcohol use, reporting reductions in mood swings and alcohol consumption. The medication-overuse headache (MOH) trial primarily investigated headache frequency but also assessed aspects of withdrawal symptom management.

Secondary outcomes across the trials included additional assessments of psychiatric symptoms, withdrawal severity, and adverse effects. Reported adverse effects included weight gain, sedation, and gastrointestinal symptoms. The risk of bias was assessed as low to moderate, with most studies reporting adequate randomization and blinding procedures. However, some trials had limitations related to allocation concealment and participant withdraw, which could influence the reliability of findings. These results provide insights into the efficacy and safety of valproic acid across different AUD subpopulations, though variations in study design and outcome measures limit direct comparisons.

### Clinical trials outcomes

The clinical trial (NCT00203528) compared divalproex ER to risperidone in 30 adults with bipolar disorder and comorbid substance use disorder in a 12-week, randomized, double-blind Phase 4 study. Participants underwent a washout period before randomization and were monitored biweekly using the CGI, GAF, and substance use assessments. Divalproex was titrated to therapeutic serum levels (80–100 mcg/mL). Although formal results were not posted, the study reported improvements in mood stability and reductions in substance use in the divalproex group, indicating potential dual benefits in this population (Le Fauve et al., 2004).

Another clinical trial (NCT01342549) was a randomized, triple-masked Phase 3 trial assessing the effectiveness of valproate versus naltrexone in 62 veterans with alcohol dependence and comorbid TBI. Conducted over 24 weeks, the trial measured time to relapse to heavy drinking, defined as consuming five or more drinks in one sitting, using the Time Line Follow Back method. Participants received either valproate (titrated up to 60 mg/kg/day) or naltrexone (50 mg/day). Results showed that valproate contributed to delayed relapse and improved mood stabilization, supporting its potential use in complex cases of AUD with psychiatric and neurological comorbidities.

The third clinical trial (NCT01760785) evaluated the effects of divalproex sodium on affective lability and alcohol use in 50 adults with a history of TBI and co-occurring alcohol misuse. This randomized, quadruple-blind trial compared divalproex sodium (750–1,250 mg/day) to placebo over an 8-week period. The primary outcome was the severity of affective lability, measured using a shortened version of the Agitated Behavior Scale (ABS). Secondary outcomes included frequency of alcohol use, assessed weekly via the Timeline Follow back method and breath alcohol testing. Results showed that valproate significantly reduced affective lability scores, and many participants also demonstrated a decrease in alcohol consumption, highlighting valproate’s potential in treating neuropsychiatric symptoms that contribute to AUD following TBI.

The fourth clinical trial (NCT00159588) was a randomized, open-label, multicenter study conducted in Norway, assessing different treatment strategies for medication-overuse headache (MOH) in 64 patients. Participants were assigned to one of three arms: abrupt withdrawal of overused medications, immediate initiation of preventive medications (including valproate), or no specific treatment (control). The primary outcome was change in headache days per month over a 5-month period. Valproate was among the preventive agents used, depending on the patient’s headache type. Although not specific to AUD, the study included valproate’s role in withdrawal management, offering indirect insights into its potential for treating alcohol-related withdrawal symptoms (Hagen et al., 2009; Hagen and Stovner, 2011).

## Discussion

The systematic analysis of clinical trials from ClinicalTrials.gov highlights the potential role of valproic acid as a therapeutic agent in managing AUD. The findings from the five included trials provide excellent understanding of the drug’s efficacy across different AUD populations, offering valuable insights into its potential benefits and limitations. One of the key findings from this analysis is that valproic acid may be particularly effective in subpopulations with coexisting psychiatric conditions, such as bipolar disorder or TBI. In these contexts, valproic acid’s mood-stabilizing properties can offer dual benefits, simultaneously addressing mood instability and reducing substance use (Salloum et al., 2005; Perugi et al., 2010). For instance, the trial comparing divalproex ER with risperidone in patients with bipolar disorder and substance abuse demonstrated that valproic acid effectively reduced mood instability and substance consumption. This suggests that the drug’s effects on GABAergic modulation.

Valproic acid’s potential therapeutic effects in patients with AUD are attributed to its multifaceted mechanism of action, which targets several neurobiological pathways implicated in addiction (Tursunov et al., 2023; Koijam et al., 2024; Lum et al., 2006). The primary pharmacological actions of valproic acid include enhancing gamma-aminobutyric acid (GABA) neurotransmission, inhibiting voltage-gated sodium channels, and modulating histone deacetylase (HDAC) activity (Mishra et al., 2021). These actions collectively contribute to its mood-stabilizing, neuroprotective, and anti-seizure properties, which may also play a role in reducing substance use and managing withdrawal symptoms (Farrokh et al., 2021; Guirguis et al., 2017). One of the key mechanisms through which valproic acid could assist individuals with AUD is through the enhancement of GABAergic activity. GABA is the primary inhibitory neurotransmitter in the central nervous system and plays a critical role in regulating neuronal excitability, stress responses, and reward pathways (Rudolph, 2022). By increasing GABA levels in the brain, valproic acid helps to dampen hyperactivity in the neural circuits associated with craving and impulsivity, which are core features of AUD (Dharavath et al., 2023). This modulation of the GABAergic system may help to reduce the reinforcing effects of addictive substances and alleviate withdrawal symptoms, thus supporting abstinence.

Valproic acid also exhibits epigenetic effects through its inhibition of histone deacetylases (HDACs), enzymes that regulate gene expression by altering chromatin structure (Sixto-Lopez et al., 2020). HDAC inhibition by valproic acid can lead to changes in the expression of genes involved in neural plasticity, stress responses, and reward processing, which are often dysregulated in AUD (Al Ameri et al., 2014). This epigenetic modulation may help to reverse some of the long-term neurobiological changes induced by alcohol, potentially restoring more normal functioning of the brain’s reward system and reducing the propensity for relapse. Furthermore, valproic acid’s impact on dopamine and glutamate neurotransmission, two key systems involved in addiction, may contribute to its therapeutic potential in AUD (Chen et al., 2006; Ueda and Willmore, 2000). By modulating these neurotransmitter systems, valproic acid can attenuate the dysregulated reward and stress circuits that drive compulsive alcohol use. This multi-target approach may make valproic acid particularly useful in managing AUD where traditional monotherapies have limited efficacy.

The use of valproic acid in the treatment of AUD and SUD requires a patient-centred approach (Celik et al., 2024). Patients with AUD frequently present with pre-existing hepatic dysfunction, necessitating baseline and routine liver function tests to monitor potential hepatotoxicity (Caputo et al., 2019). Additionally, pre-treatment screening for pancreatitis and metabolic disturbances is essential, as valproic acid has been associated with an increased risk of these conditions (Nanau and Neuman, 2013; Chapman et al., 2001). Ongoing monitoring is also required for neurological, cognitive, gastrointestinal, cardiovascular, and endocrine-related adverse effects, which have been reported in patients receiving valproic acid therapy (Singh et al., 2021). Given the complexity of valproic acid metabolism, drug interactions via CYP450 and UGT pathways should be carefully considered, particularly in patients with concurrent medication use (Shnayder et al., 2023).

This study has several limitations. The reliance on ClinicalTrials.gov only may have excluded unregistered trials, particularly those outside the United States, limiting the generalizability of the findings. While one international study (Norway) was included, most trials were U.S.-based, and the impact of unpublished data remains unknown. The heterogeneity in study populations and primary outcomes, ranging from withdrawal management to mood stabilization, restricted meta-analysis and effect size calculations, requiring a narrative synthesis instead. Variations in study design, follow-up duration, and outcome reporting may also affect reliability. While most trials used randomization and blinding, some had limitations in allocation concealment and participant withdrawal, introducing potential bias. Future research should expand on registered trials to improve external validity.

## Conclusion

Valproic acid shows potential as a treatment option for AUD, particularly in individuals with coexisting psychiatric conditions, but its role remains adjunctive rather than being prescribed as a monotherapy. The drug’s mood-stabilizing and neuroprotective properties offer a valuable addition to the existing treatment for AUD. However, its efficacy appears contingent on individual patient factors and the context of use. The findings underscore the need for personalized treatment strategies and highlight the importance of further research to explain the mechanisms through which valproic acid may benefit specific AUD subgroups. Future studies should aim to optimize its therapeutic use, assess its long-term outcomes, and explore potential combination therapies that could enhance its effectiveness in managing AUD.

## Data Availability

The original contributions presented in the study are included in the article/Supplementary Material, further inquiries can be directed to the corresponding author.
